# Excision of Joints

**Published:** 1830-07-01

**Authors:** 


### CLINICAL REVIEW.

### XLIII.

### EDINBURGH SURGICAL HOSPITAL.*

### EXCISION OF JOINTS.

SINCE November 1828, when Mr. Syme cut out a carious elbow, which he complacently remarks, "was the first time the operation was ever performed in Great Britain," he has operated in six other cases. Five of these seven cases have already been recorded in our respected Northern contemporary, and Mr. S. now furnishes the remaining two, of which we shall place an account before our readers.

*Case* 1. "Elizabeth Johnston, æt. 15, from Falkirk, entered the Hospital on the 26th of August, on account of a disease of the right elbow-joint, which had existed for six months, commenced spontaneously, and increased progressively, notwithstanding the efforts of her medical attendants. It now presented a most formidable appearance, the joint being so much swelled as to measure thirteen inches in circumference, and the arm above being reduced to little more than skin and bone, which made the enlargement seem even greater than it really was. The skin over the olecranon was extensively ulcerated, and at different places, both on the front and back parts of the joint, the probe could be passed into sinuses which extended to the bones. The limb was straight, and nearly immoveable. The discharge was profuse, the pain unceasing, and the irritation so great that the patient's health seemed rapidly sinking. It was plainly necessary to do something effectual for her relief, and both Dr. Ballingall and I, though entertaining the most favourable opinion of excision, from what we had seen of its good effects, resolved that any operation short of amputation would be inexpedient in this case, where there was such extensive disease not only of the bones, but also of the soft parts. Being, however, very averse in general to amputating the arm for caries, and feeling particular reluctance to mutilate this unfortunate girl, who was distinguished by the most amiable disposition and interesting appearance, I delayed the operation. In the course of ten days, whether it was owing to a real improvement proceeding from the free vent which had been afforded to the matter by incisions, or was merely the effect of familiarity with the appearance of the joint, I fancied that it was not so hopeless as at first believed, and resolved to make an attempt at excision.

The operation was performed in the manner formerly described, and was attended with very little difficulty, owing to the separation of the surrounding

* Edinb. Med. and Surg. Journal, No. CIII.

soft parts from the articulating bones, which had been caused by collections of matter. The olecranon was greatly expanded, and, if I may use the expression, completely rotten, so that it crumbled into fragments, which were extracted piecemeal. The radius adhered to the humerus, and was extracted along with it. Before dressing the wound, I observed that the ulnar nerve was partially divided by an oblique incision, and therefore cut it completely across, to avoid the danger of such a wound; and its extremities being then placed in contact, the integuments were stitched together. The patient did extremely well; the wound healed most kindly; the swelling of the joint subsided; she gradually regained its use; and is now, I am happy to understand, restored to perfect health. For some time after the operation, she complained of coldness and numbness in the ulnar side of the hand, but in process of time got rid of these unpleasant symptoms, probably in consequence of re-union between the extremities of the nerve."

*Case* 2. James Page, æt. 8, was admitted on the 2d of January, as a proper subject for excision of the right elbow-joint, which was much enlarged, discoloured, and stiff, with two sinuses opening on each side of the triceps, through which a probe could be passed to the bone. The operation was performed in the ordinary way on the 12th January, the wound healed kindly, and the patient is nearly ready to leave the hospital.

In the case of a lad at present in the house the operation is only delayed till the parents' consent is obtained. In another case, in which the olecranon appeared to be extensively affected with superficial caries, the bone was exposed by Mr. Syme, and the softened portion removed with a gouge; the wound is now nearly healed and the patient makes no complaint. So much for excision of the elbow-joint, and we next proceed to that of the knee. Let us hear what Mr. Syme has to say upon the subject.

"The knee-joint, so far as regards its structure, is an equally favourable subject for excision with the elbow, since there is only one articulation concerned in the disease or affected by the operation, and not a number, as is the case in the wrist or ankle. But the advantages from the operation in this situation are much more questionable than in the shoulder or elbow, since not only is there much less difference between the utility of a natural leg and a wooden one, than between that of a real and artificial arm, but doubts may even be entertained as to the probability of deriving any assistance in progressive motion from the limb, which is preserved by cutting out the knee-joint. With the exception of the two cases operated upon by Mr. Park of Liverpool, nearly fifty years ago, and the two cases lately published by Mr. Crampton of Dublin, I am not acquainted with any recorded facts to guide us in deciding this question. Each of these gentlemen lost one of their patients, but the others survived and retained limbs so useful, that the owners would not readily have exchanged them for artificial ones. Mr. Park's patient, a sailor, was able to ascend the rigging of his ship with the agility peculiar to that profession; and the woman on whom Mr. Crampton operated could walk the distance of eight or nine miles without suffering fatigue or inconvenience.

The advantages attending excision of the knee-joint over amputation in the thigh, in addition to the satisfaction of saving a limb, *and promoting the credit of surgery*, seem to me, *First*, The negative one of saving the patient from the inconvenience of resting his weight upon the *face* of a stump: *Secondly*, The positive one of preserving for him the tarsus, metatarsus, and toes, which constitute an apparatus much more efficient in protecting against the effects of concussion than any artificial one that can be constructed. Influenced by these considerations, I resolved to try the operation in some of those cases of diseased knee which so frequently result from white swelling in young subjects, and are condemned without any ceremony to amputation."

*Case*. J. Arnott, æt. 8, was admitted

Dec. 1st, with the left knee very much enlarged, and immoveably bent at an acute angle with the thigh; there were two sinuses leading to the bone on the inner side of the joint, the health was broken, and "he seemed to be devoted to *speedy destruction.*" The disease was of *three years' duration*, and had resulted from a fall on the ice.

On the 7th Mr. Syme made two incisions across the fore-part of the joint, extending from one condyle of the femur to the other, meeting at their extremities, and including the patella between them. The included integuments and patella, which was much diseased, being removed, the extremity of the femur was exposed and sawed off, but in doing this the periosteum was separated too much from the bone, and another portion of the latter was taken away. The head of the tibia was next exposed and removed by cutting pliers, one of the articular arteries tied, the wound dressed, and the limb extended by splint and bandage as far as the contraction of the hamstring muscles would allow. Exfoliation of the tibia was threatened and things at first wore a gloomy aspect, but by cautious extension and counter-extension the displaced extremities of the bones were reduced, the limb became straight, the wound in four weeks was nearly healed, and the limb is daily becoming more useful to the patient.

*Case* 2. Ann Mackintosh, æt. 7, was admitted Dec. 14th, "on account of a white-swelling of the right knee, which had existed eighteen months, and was now in its last stage;" there was a large sinus above the inner condyle, through which Mr. S. introduced his finger into the joint and felt it extensively diseased. Mr. S. performed an operation similar to that in the former case, and, as in that, the removed articulating portions of bone were extensively ulcerated and carious. "The soft parts, however, were much less swelled and less altered by the gelatinous degeneration of scrofulous action than in the boy's case; the result, therefore, was expected to be, if possible, still more satisfactory." But it was not so, for the difficulty of preventing dislocation of the bones was great, the femur, so far as it was visible, presented a bare and dead-like surface, and when Mr. Syme, in order to check more effectually the tendency to displacement, cut away about two inches more of the femur with the pliers, he was astonished to find that the bone was extensively denuded. Amputation now appeared to be the only means left, but our author waiting a little "in the expectation of nature pointing out at what part of the limb the operation ought to be performed," the patient gave Nature and Mr. Syme the slip, by dying in the interim.

"I do not think that the enemies of excision can found any thing on this case, since it would appear from reasoning, and has been in great measure proved by experience that excision of a joint is less dangerous than amputation of the limb; and the only question that can be agitated in respect to the merits of the operation in this situation concerns the utility of the limb which is preserved."

We are disposed to differ from Mr. Syme in this sentiment, for we fear that the enemies of excision and the excisor might found a good deal upon the case, and a superstructure too of a very disagreeable nature to Mr. S. We must confess that more meagre details, and more questionable observations could scarcely have been put together on the occasion. We are told in the commencement, that the case was one of "white swelling," which means any thing or nothing—we are told, that the prognosis from an operation is satisfactory, because the soft parts are comparatively little altered by the gelatinous degeneration of scrofulous action; a gelatinous degeneration of an action! We are told that because the femur is denuded farther than the finger can reach, amputation is the only chance, but that amputation is delayed till Nature points out the part of the limb, where the knife shall be applied. We know not whether Mr. Syme, like Numa, in the Roman days, may have found in the grots of the surgical hospital, an Egeria denied to his brethren, but certain we are, that Nature is by no means disposed to turn finger-post to amputating surgeons in this metropolis. We have heard indeed of a line of demarcation being formed in the progress of mortification, but in cases like Mr. Syme's we never saw, nor do we hope to see it. Finally, we must regret that the sins of omission are by no means trivial, for not a syllable is said of the general symptoms till the sudden announcement of the patient's death. It is with a case, as with a tragedy; when great and fatal events are brought about without any ostensible means, critics have ever condemned the bard, and the pit have commonly damned the play. So much for this particular case.

Mr. Syme remarks that theory and and experience combine in proving that excision of the knee-joint is less dangerous than amputation. We are not so sure of that. Mr. S. adverts to six cases, the only ones of which he has heard, and of those six, three terminated fatally. Is the mortality so great after amputations of diseased joints? We apprehend not. In making these remarks we would not be understood as entering on the discussion of the general question of excision of joints, for we really have not thought sufficiently on the subject to warrant us in pronouncing an opinion. We doubt, however, if the operation will ever become so general as Mr. Syme imagines, and we further doubt its applicability to joints like the knee. It is a painful, tedious, and we say it advisedly, unseemly operation to look at; its dangers are considerable; its advantages at the best not wholly indisputable; and we repeat that in some cases of disease of the knee-joint, when suppuration extends for some distance up the thigh, it is quite inadmissible. We believe, however, there are cases, especially of disease in the upper extremity, in which its employment may be advantageous, but these can only be determined by the experience of many and of judicious surgeons. Into their hands we commit the question.

